# Author Correction: Mitochondrial adaptor TRAK2 activates and functionally links opposing kinesin and dynein motors

**DOI:** 10.1038/s41467-026-75337-6

**Published:** 2026-07-09

**Authors:** Adam R. Fenton, Cecilia R. Petruconis, Thomas A. Jongens, Erika L. F. Holzbaur

**Affiliations:** 1 Department of Genetics, University of Pennsylvania Perelman School of Medicine, Philadelphia, PA USA; 2 Department of Physiology, University of Pennsylvania Perelman School of Medicine, Philadelphia, PA USA; 3 Cell and Molecular Biology Graduate Group, University of Pennsylvania Perelman School of Medicine, Philadelphia, PA USA; 4 Pennsylvania Muscle Institute, University of Pennsylvania Perelman School of Medicine, Philadelphia, PA USA; 5 Pharmacology Graduate Group, University of Pennsylvania Perelman School of Medicine, Philadelphia, PA USA

**Keywords:** Cytoskeletal proteins, Mitochondrial proteins, Total internal reflection microscopy, Motor protein regulation

Correction to: *Nature Communications* 10.1038/s41467-021-24862-7, published online 28 July 2021

The HA-tagged TRAK1 and TRAK2 constructs described in this paper had been accidentally switched after receipt during the generation of glycerol stocks of transformed *E. coli* strains. The plasmid labeled HA-TRAK2 (containing HA-TRAK1) was used for several experiments in the original version. The authors have repeated each of these experiments with the correct HA-TRAK2 construct and were able to validate the earlier conclusions.

To correct for the error, Figs. 1i, j, 4h, i, 5b–e and associated statistical information in figure legends, Supplementary Figs. 4a, b, 6a, b and Supplementary Videos 2, 3 have been replaced. Text in the fourth paragraph of the Results, the third paragraph of the Results “Kinesin-1 and dynein–dynactin promote TRAK2 transport by the opposing motor” subsection and the second paragraph of the Methods have been amended. For comparison, the original, uncorrected article is available as Supplementary Information accompanying this amendment. The changes are made in the HTML and PDF versions of the article.

## Supplementary information

Original article

