# Supplementary material for: Mitochondrial adaptor TRAK2 activates and functionally links opposing kinesin and dynein motors
Source: Nat Commun. 2021 Jul 28;12:4578. doi: 10.1038/s41467-021-24862-7 (PMC8319186; doi:10.1038/s41467-021-24862-7)
Supplement: Supplementary file 1 — Supplementary Information [file 41467_2021_24862_MOESM1_ESM.pdf]

Mitochondrial adaptor TRAK2 activates and functionally links opposing kinesin and dynein motors

Adam R. Fenton[1-4], Cecilia R. Petruconis[2,4,5], Thomas A. Jongens[1,3]*, Erika L. F. Holzbaur[2-4]*

Supplementary Information

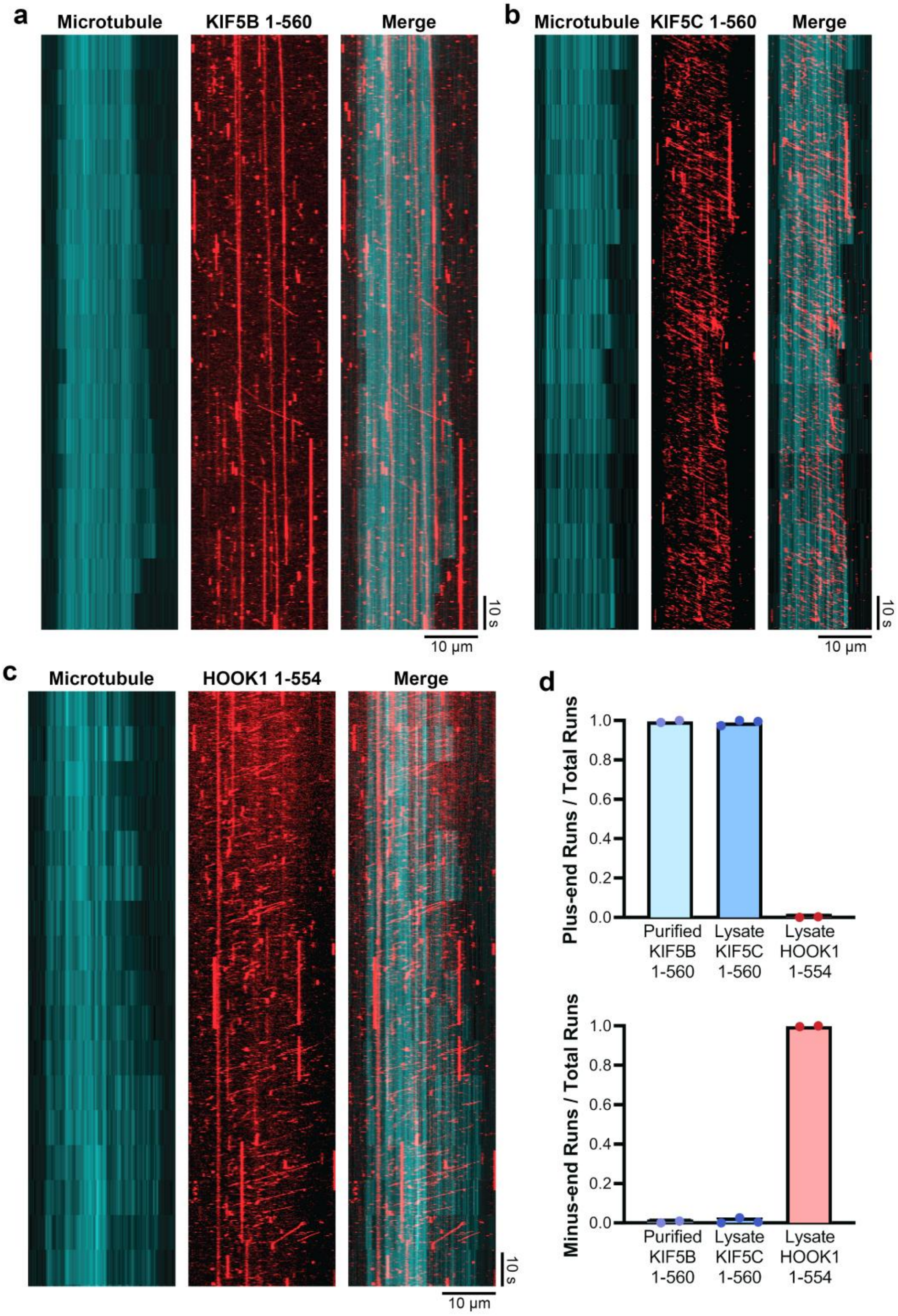

Supplementary Figure 1 Motility of kinesin-1 and dynein on dynamic microtubules. **a** Motility of 1 nM purified KIF5B(1–560)-Halo on dynamic microtubules. Left panel shows full kymograph of dynamic microtubule channel. Middle panel shows full kymograph of KIF5B 1-560 channel. **b** Same as **a**, but for KIF5C(1-560)-Halo present in COS-7 cell lysate. **c** Same as **a**, but for Halo-HOOK1(1-554) present in COS-7 cell lysate. **d** Quantification of transport direction along dynamic microtubules for KIF5B 1-560, KIF5C 1-560, and HOOK1 1-554 constructs. The bars represent the mean (n = 170 runs from 2 experiments for purified KIF5B 1-560, 1060 runs from 3 experiments for lysate KIF5C 1-560, and 678 runs from 2 experiments for lysate HOOK1 1-554).

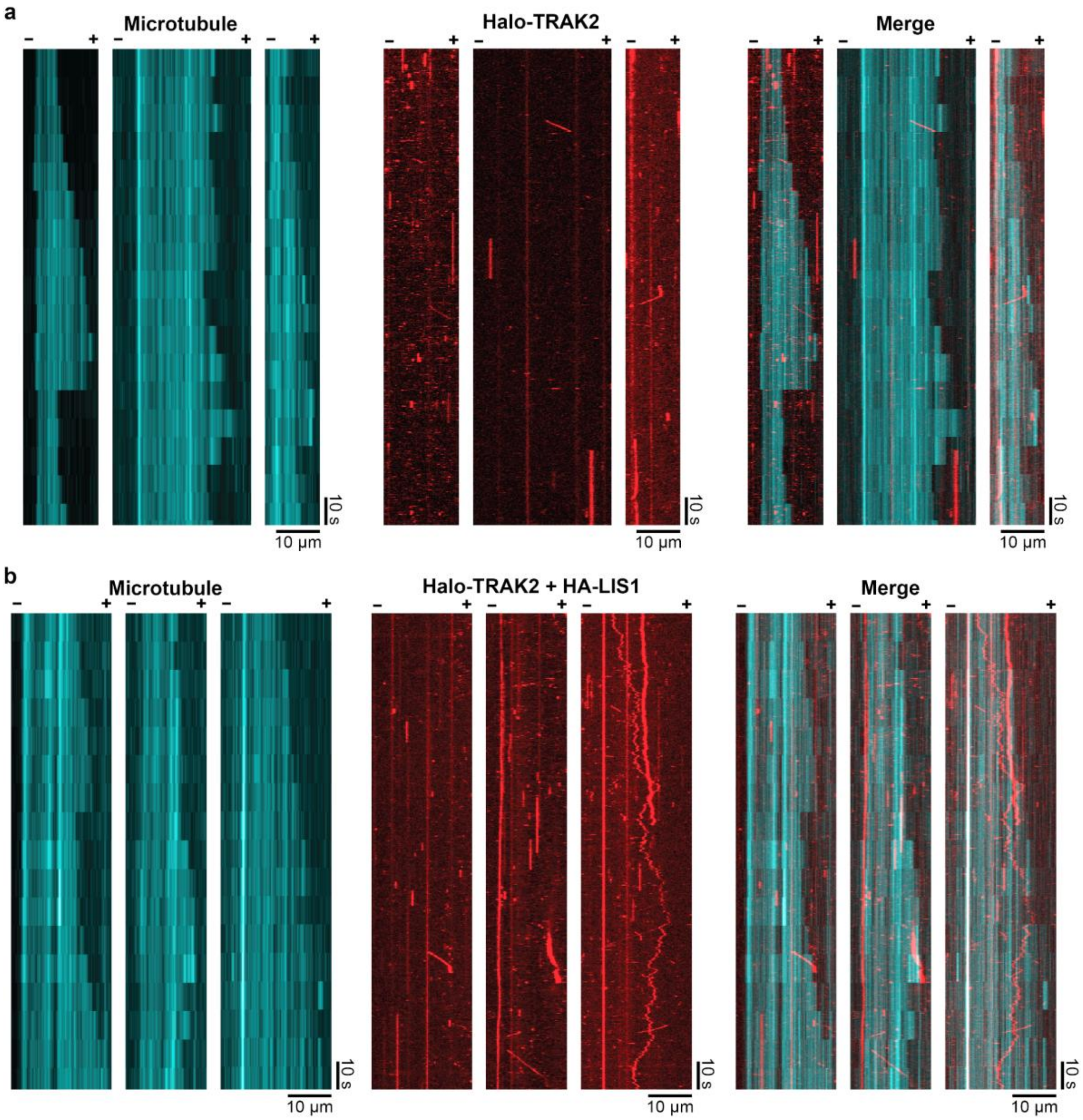

Supplementary Figure 2 Halo-TRAK2 motility on dynamic microtubules. **a** Halo-TRAK2 motility on dynamic microtubules. The left panel shows three examples of 3-minute kymographs of the dynamic microtubule channel. The microtubule plus-end is evident from the greater growth and catastrophe rate at the right of each kymograph. The middle panel shows corresponding 3-minute kymographs of the Halo-TRAK2 channel for the same microtubules. The right panel shows a merge of the other two channels. **b** Same as **a**, but in the presence of exogenously expressed HA-LIS1.

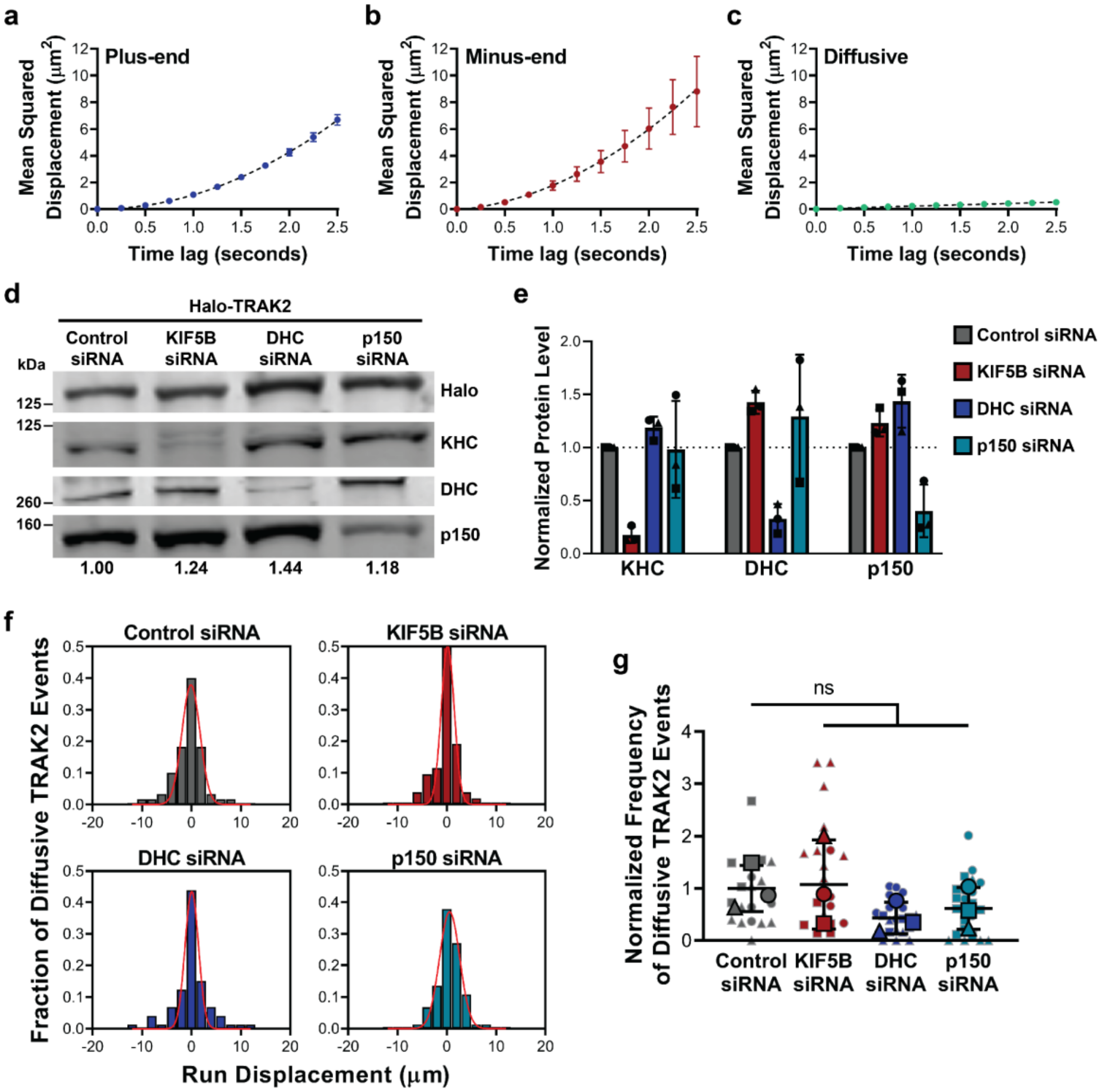

Supplementary Figure 3 The bidirectional motility of TRAK2 is consistent with diffusion. **a** Mean-squared displacement (MSD) analysis of unidirectional TRAK2 movement to the microtubule plus-end is processive ($MSD=v^2t^2$). **b** MSD analysis of unidirectional TRAK2 movement to the microtubule minus-end is processive ($MSD=v^2t^2$). **c** MSD analysis of TRAK2 bidirectional motility fits a diffusive state (MSD=2Dt). Error bars indicate s.e.m. (n = 15 events per condition). **d** Western blot verification of siRNA knockdown in Halo-TRAK2 cell lysates used for single-molecule experiments. Blots were probed for Halo-TRAK2, kinesin heavy chain (KHC), dynein heavy chain (DHC), and $p150^{Glued}$. Relative protein levels from total protein stain are shown for each condition. **e** Quantification of protein levels for KHC, DHC, and p150 in siRNA knockdown experiments. The level of each protein is normalized to the level of the control siRNA condition for each replicate. Data are mean ± s.d. with points shaped according to experimental replicate, (n = 3 independent experiments). **f** Distribution of run displacement for TRAK2 diffusive movement upon knockdown with a control, KIF5B, dynein heavy chain (DHC), or p150 siRNA (n = 60, 52, 70, and 73 events, respectively). Negative displacement indicates movement to the microtubule minus-end. The red line represents a fit to a Gaussian distribution. **g** Normalized frequency of TRAK2 diffusion upon motor knockdown. Data points represent the frequency of TRAK2 motility per video normalized to the average frequency of control siRNA events and are shaped according to experimental replicate, with smaller points representing diffusive frequency per video. The center line and bars represent mean ± s.d., (n = 19 videos for control siRNA, 19 videos for KIF5B siRNA, 21 videos for DHC siRNA, and 23 videos for p150 siRNA, 3 independent experiments). ns, not significant (one-way ANOVA with Dunnett's multiple comparisons test).

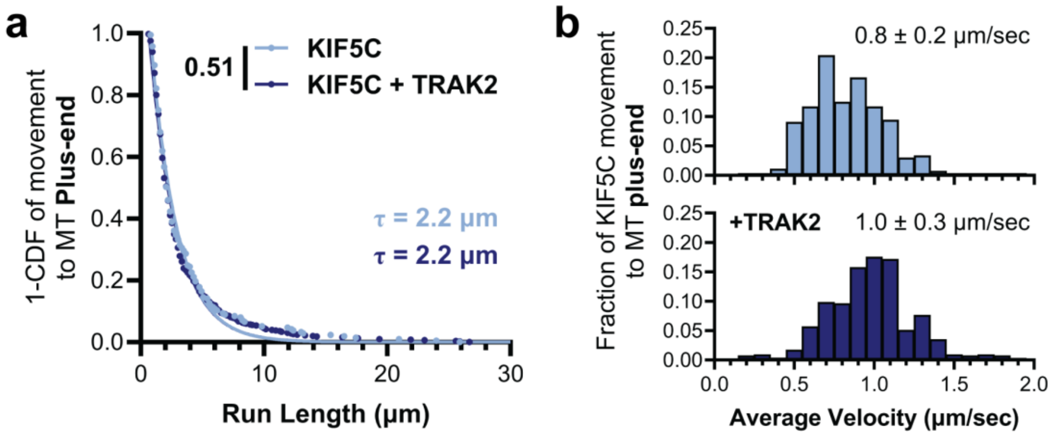

Supplementary Figure 4 TRAK2 overexpression increases KIF5C velocity. **a** Inverse cumulative distribution functions (CDF) of run lengths for KIF5C transport to the microtubule plus-end in the presence or absence of exogenous TRAK2. The curves represent single exponential decay fits, with the decay constants for each indicated above (n = 264 events without TRAK2 and 506 events with TRAK2). p-value = 0.51 from two-tailed Mann-Whitney U test. **b** Histogram of velocity distributions for KIF5C transport to the microtubule plus-end in the presence or absence of exogenous TRAK2. The values are mean ± s.d.

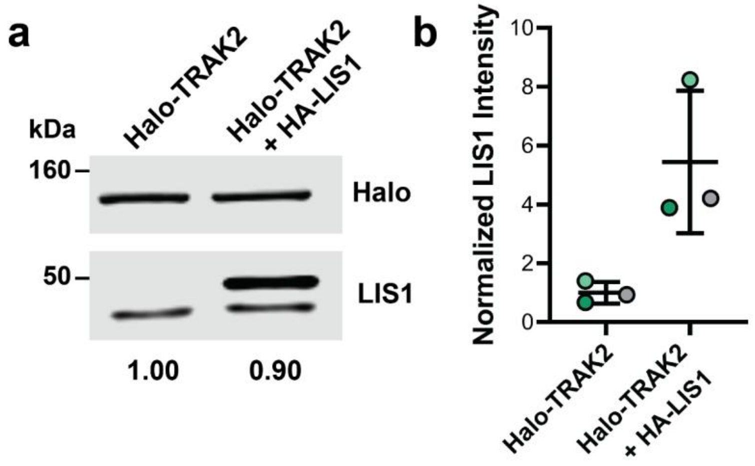

Supplementary Figure 5 Overexpression of LIS1 in samples from single-molecule experiments. **a** Representative Western blot showing the expression of Halo-TRAK2 and LIS1 in cell lysates used for single-molecule experiments. Relative protein levels from total protein stain are shown for each condition. **b** Quantification of LIS1 intensity in cell lysates normalized to total protein levels. Bars represent mean ± s.d. from 3 independent experiments.

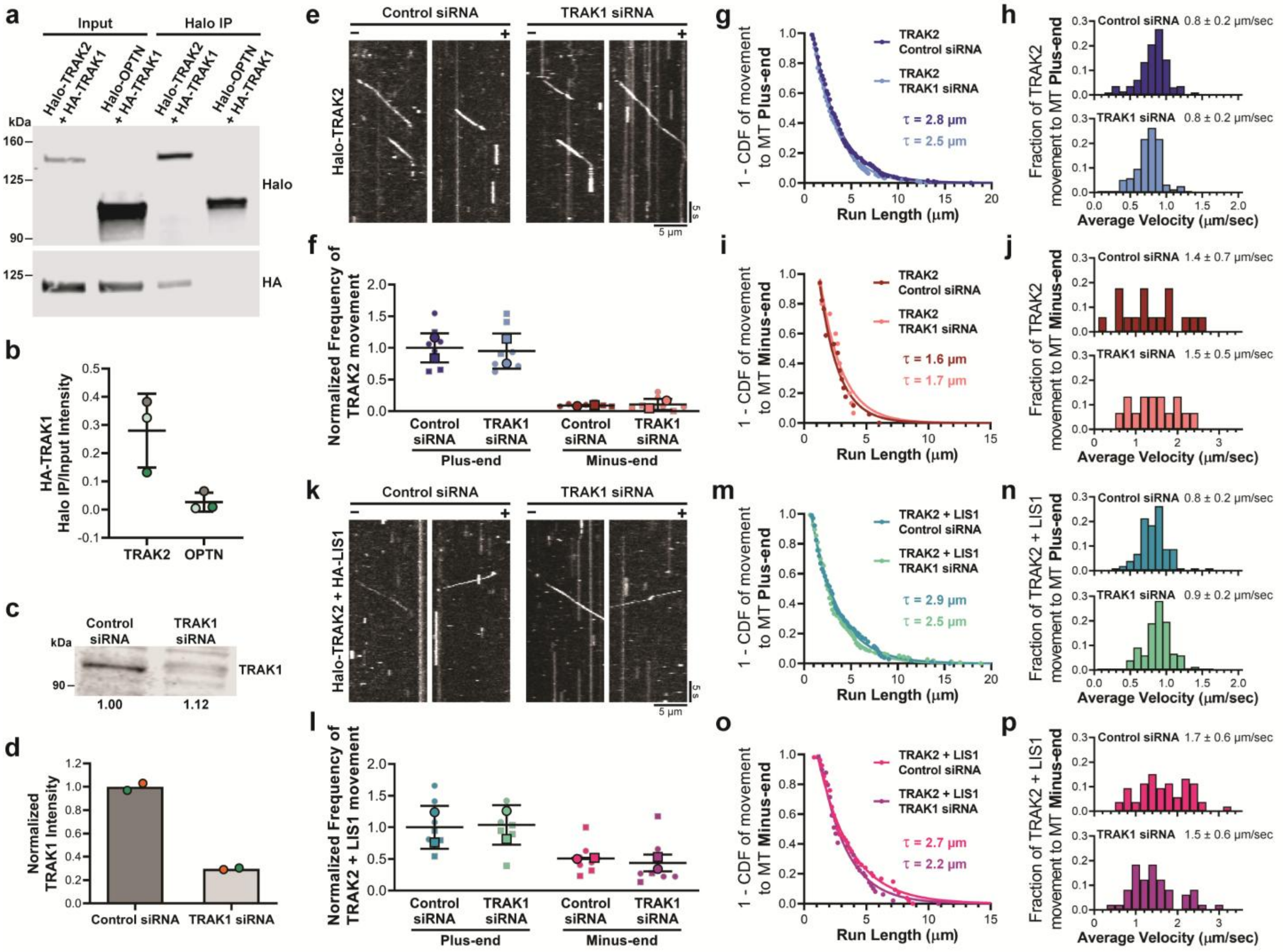

Supplementary Figure 6 TRAK1 interacts with TRAK2, but depletion of endogenous TRAK1 does not alter TRAK2 transport in single-molecule experiments. **a** Immunoprecipitation using a Halo antibody of extracts from COS-7 cells transfected with HA-TRAK1 and Halo-tagged TRAK2 or negative control Optineurin (OPTN). **b** Quantification of co-immunoprecipitation of HA-TRAK1 with Halo-tagged constructs. The center line and bars represent mean ± s.d. with data points color-coded according to experimental replicate (n = 3 experiments). **c** Western blot verification for siRNA knockdown of TRAK1. Relative protein levels from total protein stain are shown for each condition. **d** Quantification of TRAK1 knockdown. Bars represent the mean with data points color-coded according to experimental replicate (n = 2 experiments).**e** Representative kymographs showing how siRNA knockdown of TRAK1 affects TRAK2 motility along MTs. **f** Normalized frequency of TRAK2 motile events with control siRNA or TRAK1 siRNA. Data points are shaped according to experimental replicate, with smaller points representing TRAK2 frequency per video. The center line and bars represent the mean ± s.d., (n = 8 videos per condition, 2 experiments). **g-j** Inverse cumulative distribution functions (CDF) of run length and histogram distributions of velocity for TRAK2 transport to either microtubule end (n = 194 plus-end events with control siRNA, 156 plus-end events with TRAK1 siRNA, 17 minus-end events with control siRNA, and 15 minus-end events with TRAK1 siRNA). The curves in **g,i** represent single exponential decay fits with decay constants indicated above. In **h,j** the mean ± s.d. is shown. **k** Representative kymographs showing how siRNA knockdown of TRAK1 affects TRAK2 motility along MTs when HA-LIS1 is expressed. **l** Same as **f**, but with HA-LIS1 expressed (n = 8 videos with control siRNA and 8 videos with TRAK1 siRNA, 2 independent experiments). **m-p** Same as **g-j**, but with HA-LIS1 expressed (n = 103 plus-end events with control siRNA, 153 plus-end events with TRAK1 siRNA, 54 minus-end events with control siRNA, and 49 minus-end events with TRAK1 siRNA).

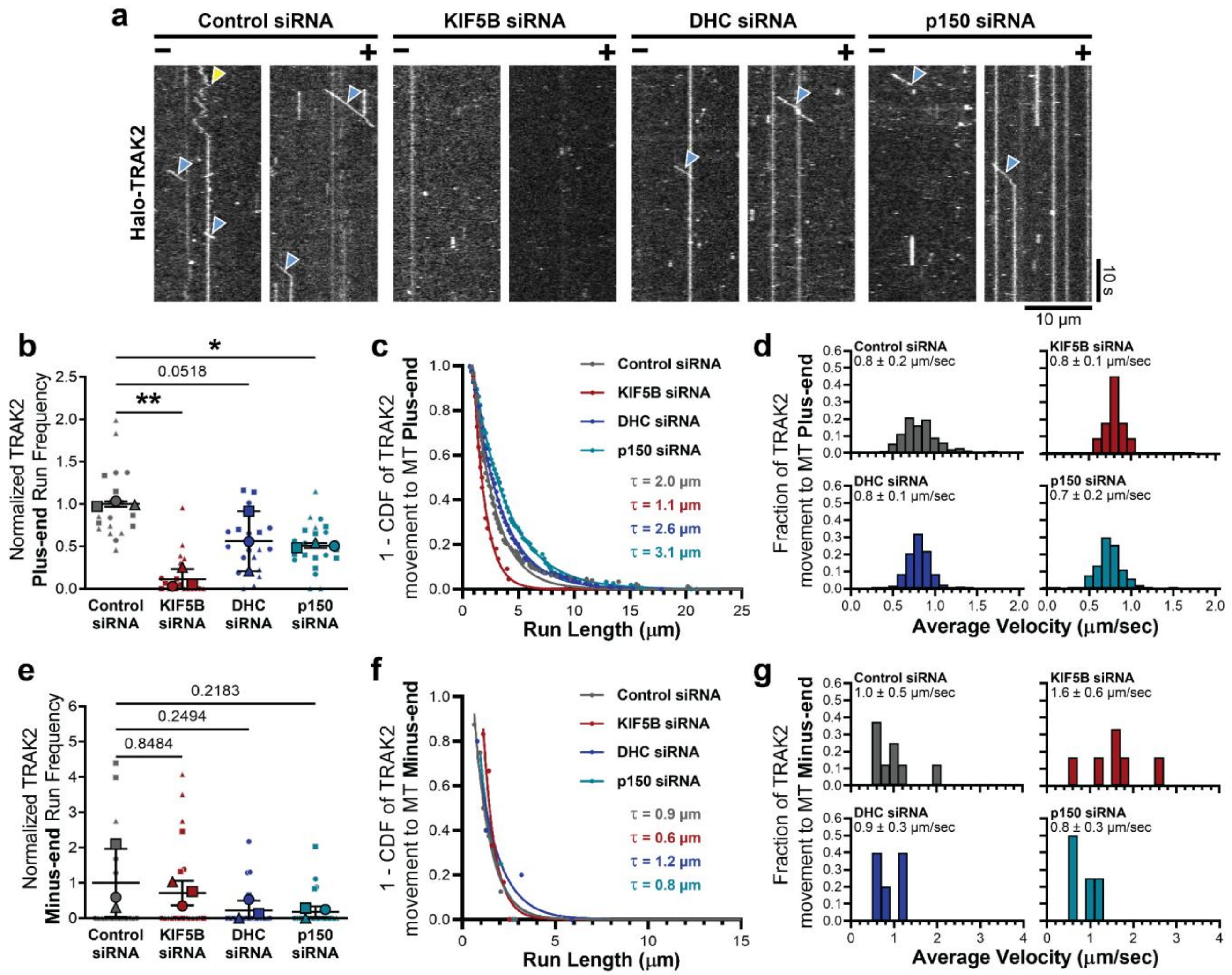

Supplementary Figure 7 Dynein-dynactin promotes plus-end-directed TRAK2 transport in the absence of exogenous LIS1. **a** Representative kymographs showing how siRNA knockdown of KIF5B, dynein heavy chain (DHC), or p150^{Glued} (p150) affects TRAK2 motility along MTs. Blue and yellow arrows indicate plus-end and diffusive TRAK2 movement, respectively. **b** Normalized frequency of TRAK2 transport to the MT plus-end upon motor knockdown. Data points represent the frequency of TRAK2 motility per video normalized to the average frequency of control siRNA events. The center line and bars represent mean ± s.d., (n = 19 for control siRNA, 21 for KIF5B siRNA, 25 for DHC siRNA, and 23 for p150 siRNA, 3 independent experiments). Exact p-values are shown when p > 0.05; *, p = 0.0319; **, p = 0.0011 (one-way ANOVA with Dunnett's multiple comparisons test). **c,d** Inverse cumulative distribution functions (CDF) of run length and histogram distributions of velocity for TRAK2 transport to the microtubule plus-end (n = 177 events for control siRNA, 22 events for KIF5B siRNA, 351 events for DHC siRNA, and 153 events for p150 siRNA). The curves in **c** represent single exponential decay fits with decay constants indicated above. The values in **d** are mean ± s.d. **e** Same as **b**, but for TRAK2 transport to the microtubule minus-end. Exact p-values are shown when p > 0.05 (one-way ANOVA with Dunnett's multiple comparisons test). **f,g** Same as **c,d**, but for TRAK2 transport to the microtubule minus-end (n = 8 events for control siRNA, 6 events for KIF5B siRNA, 5 events for DHC siRNA, and 4 events for p150 siRNA).

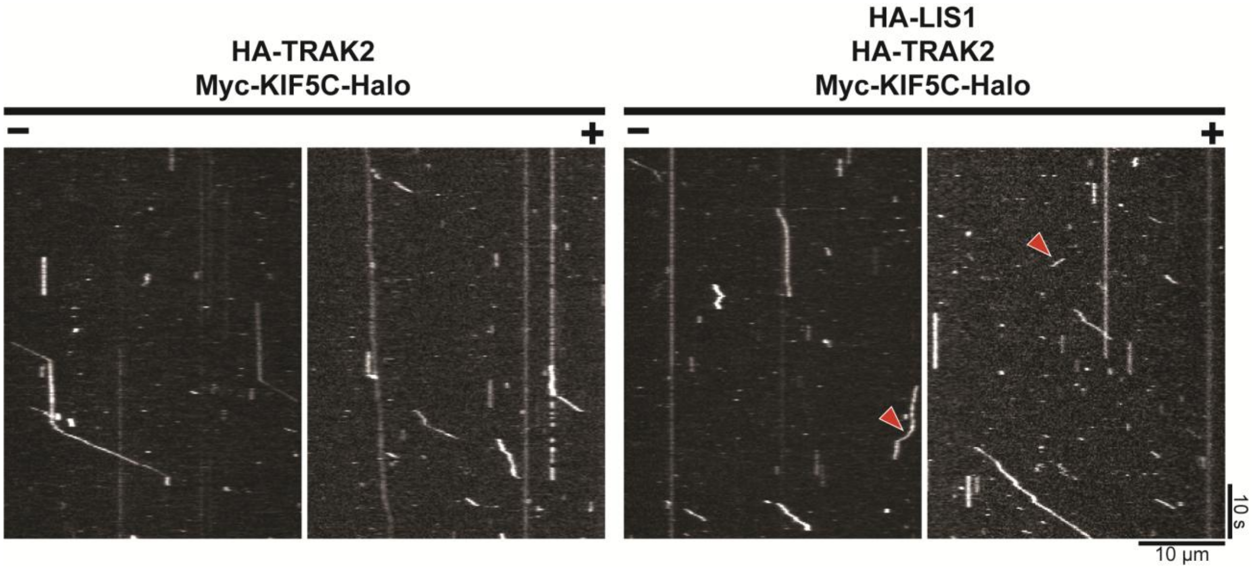

Supplementary Figure 8 LIS1 promotes minus-end-directed transport of kinesin-1 in the presence of TRAK2. Representative kymographs show unidirectional KIF5C transport toward the microtubule plus-end in the presence of TRAK2 alone and instances of minus-end-directed KIF5C transport in the presence of TRAK2 and LIS1. Minus-end-directed KIF5C runs are indicated with red arrows.

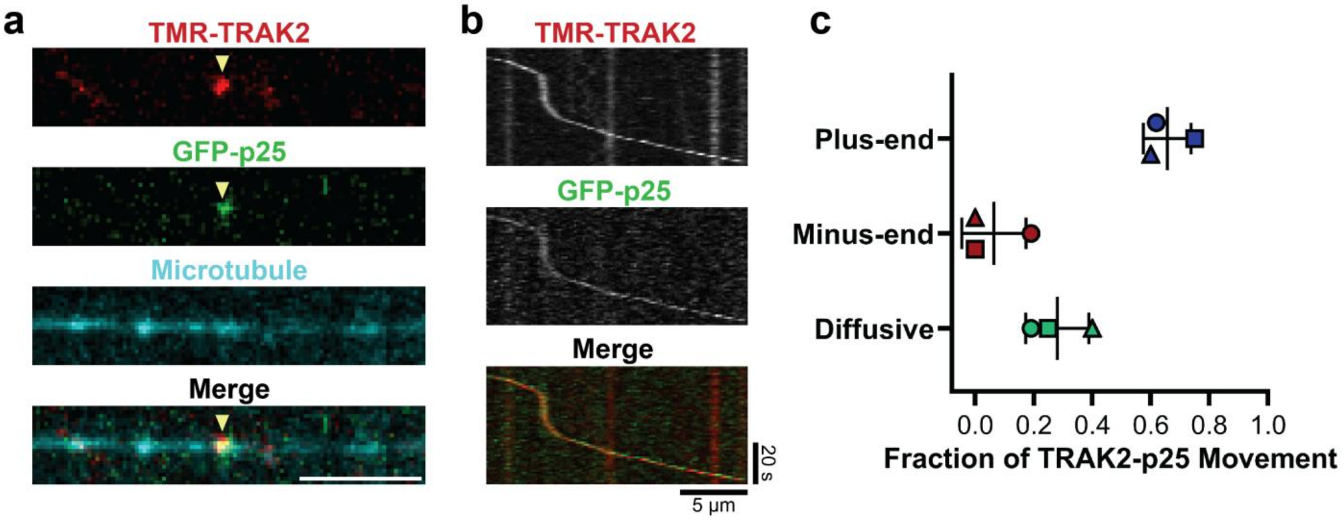

Supplementary Figure 9 TRAK2-p25 co-complexes move processively toward either microtubule end. **a** Example still showing co-localization of TRAK2 and the p25 subunit of dynactin on a microtubule. Scale bar is 5 μm. **b** Representative kymographs showing processive co-migration of TRAK2 with p25. **c** Fraction of occurrence for plus-end-directed, minus-end-directed, and diffusive movement of TRAK2-p25 co-complexes. Data points are shaped according to experimental replicate. The center line and bars are the mean ± s.d. from 3 independent experiments.

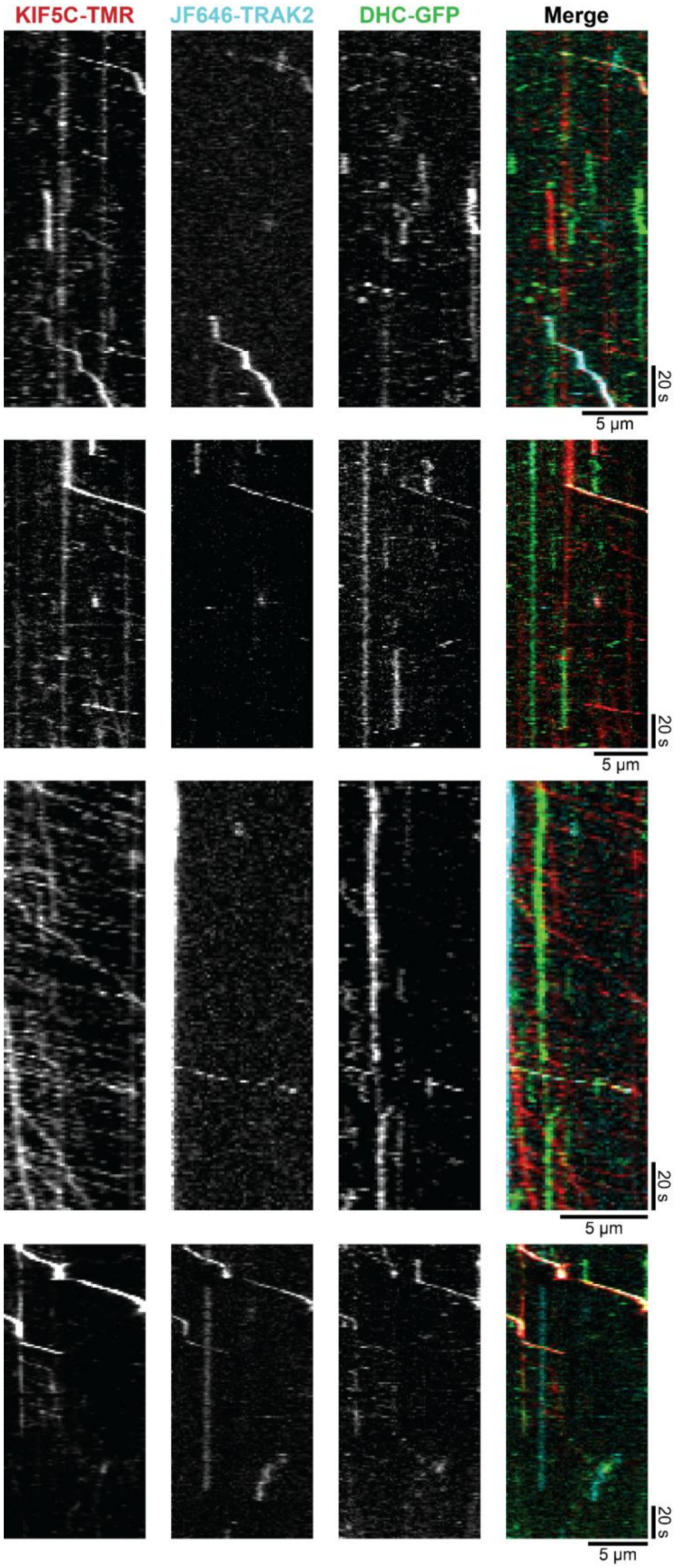

Supplementary Figure 10 TRAK2-KIF5C-DHC co-complexes move processively toward the microtubule plus-end. A panel of representative kymographs show unidirectional co-complex transport toward the microtubule plus-end. Kymographs are oriented with the microtubule plus-end on the right, as shown by the trajectories of complexes containing KIF5C alone.
