# Supplementary material for: Mitochondrial adaptor TRAK2 activates and functionally links opposing kinesin and dynein motors
Source: Nat Commun. 2021 Jul 28;12:4578. doi: 10.1038/s41467-021-24862-7 (PMC8319186; doi:10.1038/s41467-021-24862-7)
Supplement: Supplementary file 3 — Description of Additional Supplementary Files [file 41467_2021_24862_MOESM3_ESM.pdf]

Description of Additional Supplementary Files

File Name: Supplementary Movie 1
Description: TRAK2 moves processively to the microtubule plus-end. Representative video showing a single TMR-Halo-TRAK2 particle (red) moving toward the dynamically growing plus-end of a surfaceattached dynamic MT (cyan). The MT plus-end (right) is evident by the faster growth rate. Scale bar is 5 µm. Stopwatch shows time in minutes: seconds.

File Name: Supplementary Movie 2
Description: KIF5C displays infrequent movement along microtubules when expressed alone. Representative video showing a single Myc-KIF5C-Halo-TMR particle (red) moving along a surface-attached dynamic MT (cyan). Scale bar is 5 µm. Stopwatch shows time in minutes: seconds.

File Name: Supplementary Movie 3
Description: TRAK2 induces robust KIF5C movement along microtubules. Representative video showing many Myc-KIF5C-Halo-TMR particles (red) moving along a surface-attached dynamic MT (cyan) when HA-TRAK2 is co-expressed. Scale bar is 5 µm. Stopwatch shows time in minutes: seconds.

File Name: Supplementary Movie 4
Description: TRAK2 moves processively to the microtubule minus-end in the presence of exogenous HA-LIS1. Representative video showing a single TMR-Halo-TRAK2 particle (red) moving toward the minusend of a surface-attached dynamic MT (cyan) when HA-LIS1 is expressed. The MT plus-end (right) is evident by the faster growth rate. Scale bar is 5 µm. Stopwatch shows time in minutes: seconds.

File Name: Supplementary Movie 5
Description: TRAK2 switches directions during processive movement. Representative video showing a single TMR-Halo-TRAK2 particle (red) reversing direction while moving processively along a microtubule (cyan). Scale bar is 5 µm. Stopwatch shows time in minutes: seconds.

File Name: Supplementary Movie 6
Description: TRAK2, kinesin-1, and dynein co-migrate as a complex. Representative video with JF646-SNAP-TRAK2 (top channel, cyan), Myc-KIF5C-Halo-TMR (second channel, red), DHC-GFP (third channel, green), and merge (bottom channel) showing these components comigrating as a single complex along an unlabeled microtubule. Scale bar is 2 µm. The movie is 36 seconds of real time.
