# Supplementary material for: Mitochondrial adaptor TRAK2 activates and functionally links opposing kinesin and dynein motors
Source: Nat Commun. 2021 Jul 28;12:4578. doi: 10.1038/s41467-021-24862-7 (PMC8319186; doi:10.1038/s41467-021-24862-7)
Supplement: Supplementary file 10 — Reporting Summary [file 41467_2021_24862_MOESM10_ESM.pdf]

nature research

Corresponding author(s): Thomas Jongens, Erika Holzbaur

Last updated by author(s): Jun 29, 2021

Reporting Summary

Nature Research wishes to improve the reproducibility of the work that we publish. This form provides structure for consistency and transparency in reporting. For further information on Nature Research policies, see our Editorial Policies and the Editorial Policy Checklist.

Statistics

For all statistical analyses, confirm that the following items are present in the figure legend, table legend, main text, or Methods section.

n/a	Confirmed	
☐	☒	The exact sample size (*n*) for each experimental group/condition, given as a discrete number and unit of measurement
☐	☒	A statement on whether measurements were taken from distinct samples or whether the same sample was measured repeatedly
☐	☒	The statistical test(s) used AND whether they are one- or two-sided<br>*Only common tests should be described solely by name; describe more complex techniques in the Methods section.*
☒	☐	A description of all covariates tested
☐	☒	A description of any assumptions or corrections, such as tests of normality and adjustment for multiple comparisons
☐	☒	A full description of the statistical parameters including central tendency (e.g. means) or other basic estimates (e.g. regression coefficient) AND variation (e.g. standard deviation) or associated estimates of uncertainty (e.g. confidence intervals)
☐	☒	For null hypothesis testing, the test statistic (e.g. *F*, *t*, *r*) with confidence intervals, effect sizes, degrees of freedom and *P* value noted<br>*Give P values as exact values whenever suitable.*
☒	☐	For Bayesian analysis, information on the choice of priors and Markov chain Monte Carlo settings
☒	☐	For hierarchical and complex designs, identification of the appropriate level for tests and full reporting of outcomes
☒	☐	Estimates of effect sizes (e.g. Cohen's *d*, Pearson's *r*), indicating how they were calculated

Our web collection on statistics for biologists contains articles on many of the points above.

Software and code

Policy information about availability of computer code

Data collection	Single-molecule data were collected using Volocity software (Perkin Elmer) version 6.4.0. Western blot data were collected using Image Studio (LI-COR) version 5.2.
Data analysis	Data were analyzed using FIJI 2.0.0-rc-67/1.53d, GraphPad Prism version 9.1.0, and R version 4.0.3.

For manuscripts utilizing custom algorithms or software that are central to the research but not yet described in published literature, software must be made available to editors and reviewers. We strongly encourage code deposition in a community repository (e.g. GitHub). See the Nature Research guidelines for submitting code & software for further information.

Data

Policy information about availability of data

All manuscripts must include a data availability statement. This statement should provide the following information, where applicable:

- Accession codes, unique identifiers, or web links for publicly available datasets
- A list of figures that have associated raw data
- A description of any restrictions on data availability

No large datasets were generated or analyzed in this study. The source data underlying all figures and supplementary figures are provided as a Source data file. Data supporting the findings of this manuscript are also available from the corresponding authors upon request.

Field-specific reporting

Please select the one below that is the best fit for your research. If you are not sure, read the appropriate sections before making your selection.

☒ Life sciences ☐ Behavioural & social sciences ☐ Ecological, evolutionary & environmental sciences

For a reference copy of the document with all sections, see nature.com/documents/nr-reporting-summary-flat.pdf

Life sciences study design

All studies must disclose on these points even when the disclosure is negative.

Sample size	For single-molecule experiments, no pre-specification of sample size was used in the experimental design. Sample sizes for in vitro motility experiments were determined by the number of molecules and microtubules present per field of view in each flow chamber. Typically, 3 to 5 fields of view were imaged per flow chamber, increasing the sample size. The sample sizes in our motility experiments were determined based on previous experiences and relevant publications (Olenick et al. 2016, JBC; Ayloo et al. 2014, Nat. Comm.).<br>Immunoprecipitation experiments were performed three times, except for Supplementary Figure 6a, which was performed twice.<br>Pelleting experiments were performed five times due to the high level of variability inherent to this assay.
Data exclusions	In single-molecule motility experiments, molecules with runs less than 632 nm were excluded from analysis as these short runs cannot be distinguished from noise given the sensitivity of our system. This cutoff was determined before the experiments in this study were performed.
Replication	All experiments were performed a minimum of 3 times, with the exception of Supplementary Fig. 1 and Supplementary Fig. 6, which contain experiments performed 2 times. Details of the number of independent experiments are given in the figure legends.
Randomization	For each experiment, cultured cells were transfected in parallel with all dishes of cells randomly allocated into groups. For in vitro motility experiments, random field of views were chosen for analysis. Microtubules were randomly chosen from the field of view for analysis.
Blinding	For in vitro motility experiments, videos were acquired with knowledge of experimental condition. The investigators were then blinded to experimental conditions for analysis of videos. Blinding was not performed for immunoprecipitation and pelleting experiments as knowledge of experimental conditions is required for western blotting.

Reporting for specific materials, systems and methods

We require information from authors about some types of materials, experimental systems and methods used in many studies. Here, indicate whether each material, system or method listed is relevant to your study. If you are not sure if a list item applies to your research, read the appropriate section before selecting a response.

Materials & experimental systems

n/a	Involved in the study	
☐	☒	Antibodies
☐	☒	Eukaryotic cell lines
☒	☐	Palaeontology and archaeology
☒	☐	Animals and other organisms
☒	☐	Human research participants
☒	☐	Clinical data
☒	☐	Dual use research of concern

Methods

n/a	Involved in the study	
☒	☐	ChIP-seq
☒	☐	Flow cytometry
☒	☐	MRI-based neuroimaging

Antibodies

Antibodies used	The following primary antibodies were used in this study: rabbit anti-HaloTag (Promega, G9281), mouse anti-p150Glued (BD Transduction Laboratories, 610474), mouse anti-Kinesin Heavy Chain (Millipore, MAB 1614, clone H2), rabbit anti-Dynein Heavy Chain (Santa Cruz Biotechnology, R-325), mouse anti-FLAG (Sigma, F4042), mouse anti-Myc (Sigma, R950-25), mouse anti-HA (Covance, 16B12), mouse anti-GFP (Abcam, ab1218), rabbit anti-LIS1 (Abcam, ab109630), and rabbit anti-TRAK1 (Thermo, PA5-44180). Secondary antibodies included the following: IRDye 800CW donkey anti-rabbit IgG (LI-COR, 926-32213), IRDye 680RD donkey anti-rabbit IgG (LI-COR, 926-68073), and IRDye 800CW donkey anti-mouse IgG (LI-COR, 926-32212).
Validation	The rabbit anti-HaloTag, mouse anti-Myc, mouse anti-FLAG, mouse anti-HA, and rabbit anti-LIS1 antibodies were validated for western blotting by overexpression of tagged constructs in COS-7 cells (Fig. 3i, Fig. 5b,d, Supplementary Fig. 5a). The mouse anti-Kinesin Heavy Chain, rabbit anti-Dynein Heavy Chain, mouse anti-p150Glued, and rabbit anti-TRAK1 antibodies were validated for western blotting by siRNA knockdown in COS-7 cells (Fig. 4h, Supplementary Fig. 3d, Supplementary Fig. 6c). The rabbit anti-HaloTag, mouse anti-GFP, and mouse anti-FLAG antibodies were previously validated for immunoprecipitation (Fu et al. 2013, JCB; Cason et al. 2021, JCB).

Eukaryotic cell lines

Policy information about cell lines

Cell line source(s)	COS-7 cells (CRL-1651) were purchased from the American Type Culture Collection. HeLa cells stably expressing GFP-tagged dynein heavy chain were a gift from Tony Hyman (Max Planck Institute for Molecular Cell Biology and Genetics) and generated in Poser et al. 2008, Nature Methods.
Authentication	COS-7 and DHC-GFP HeLa cells were not authenticated.
Mycoplasma contamination	The cell lines used here are tested annually for mycoplasma contamination. These cells tested negative for mycoplasma contamination by PCR.
Commonly misidentified lines (See ICLAC register)	No commonly misidentified cell lines were used in this study.
